# SpatialSim: Recognizing Spatial Configurations of Objects With Graph Neural Networks

**DOI:** 10.3389/frai.2021.782081

**Published:** 2022-01-26

**Authors:** Laetitia Teodorescu, Katja Hofmann, Pierre-Yves Oudeyer

**Affiliations:** ^1^Flowers Team, Inria Bordeaux, Talence, France; ^2^Microsoft Research, Cambridge, United Kingdom

**Keywords:** graph neural net, neural networks, similarity learning, structured representation, machine learning, artificial intelligence, spatial reasoning

## Abstract

An embodied, autonomous agent able to set its own goals has to possess geometrical reasoning abilities for judging whether its goals have been achieved, namely it should be able to identify and discriminate classes of configurations of objects, irrespective of its point of view on the scene. However, this problem has received little attention so far in the deep learning literature. In this paper we make two key contributions. First, we propose SpatialSim (Spatial Similarity), a novel geometrical reasoning diagnostic dataset, and argue that progress on this benchmark would allow for diagnosing more principled approaches to this problem. This benchmark is composed of two tasks: “Identification” and “Discrimination,” each one instantiated in increasing levels of difficulty. Secondly, we validate that relational inductive biases—exhibited by fully-connected message-passing Graph Neural Networks (MPGNNs)—are instrumental to solve those tasks, and show their advantages over less relational baselines such as Deep Sets and unstructured models such as Multi-Layer Perceptrons. We additionally showcase the failure of high-capacity CNNs on the hard Discrimination task. Finally, we highlight the current limits of GNNs in both tasks.

## 1. Introduction

Building autonomous agents that can explore their environment and build an open-ended repertoire of skills is a long-standing goal of developmental robotics and artificial intelligence in general. To this end, one option that has been explored in the literature is autotelic agents: agents that can set their own goals and learn to reach them. Within the deep learning community, this has taken the form of Deep Reinforcement Learning agents that take a goal—expressed for instance as a target state of the world, a sentence in some language, or some other representation—as input additionally to the observation; these agents are able to alter their behaviors in light of the provided goal to execute it in the environment. Crucial to the development of such agents, especially from a developmental perspective, is the learning of a goal-achievement function (or reward function) that measures how close the agent is to reaching its goal (Bahdanau et al., 2019; Colas et al., 2020). This warrants the independent study of such goal-achievement functions. One of the design choices this implies is the chosen representation for the goal, observed state and the associated network architecture for the reward and policy. Since this representation is crucial for performance and robustness of the networks, a principled approach would be representing these states and goals in a cognitively plausible way.

The world appears to us as immediately organized into collections of objects, arranged together in natural scenes. These independent entities are the support for mental manipulation and language, and can be processed separately and in parallel (Kahneman et al., 1992; Pylyshyn, 2007; Green and Quilty-Dunn, 2017). These objects are themselves composed of constituent elements whose precise arrangement in space determines their properties. To implement this kind of structure into neural networks, there is a strong emerging movement that advocates for the use of more structured models (Lake et al., 2016; Battaglia et al., 2018), in particular for using models displaying *relational inductive biases* (Battaglia et al., 2018). Inductive biases are constraints that bias the convergence of function approximators toward a particular kind of function. In deep learning, the most common form of inductive bias is given by the architecture of the model. *Relational inductive biases* refer to architectures supporting the processing of data composed of separate objects, described in the same space, and their relations. Graph Neural Networks (GNNs) (Gori et al., 2005; Kipf and Welling, 2016; Gilmer et al., 2017), function approximators operating on graph-structured input (that can return graph, set, or scalar output) naturally implement these inductive biases; this is so because the computation they perform is a composition of 1) independent but similar operations on object representations and 2) of binary functions taking pairs of objects (edge computation, representing the relational part). Models based on these ideas have achieved substantial progress compared to unstructured methods in recent years on graph-based data or on more general scenes, whether it is by considering the input as sets of objects in a shared space (Qi et al., 2016; Zaheer et al., 2017) or by explicitly considering the relations between objects (Battaglia et al., 2016; Kipf et al., 2019).

Objects and their relations thus seems like a natural representation for states of the world, and thus, by extension, for goals a goal-conditioned agent has to reach. This representation lends itself to geometrical, or spatial, reasoning. Spatial reasoning implies processing configurations of objects and their precise relations in space to judge for instance whether a particular structure of blocks is stable or not (Hamrick et al., 2018), a task that requires fine-grained reasoning over the shapes, orientations and positions of the blocks. These kinds of representations can be acquired in an unsupervised manner directly from the observations (Burgess et al., 2019; Greff et al., 2020; Locatello et al., 2020), which makes them appealing in a context where the agent has to abstract away some of the information in its goal (thus it cannot represent its goal as a target image, it is too low-level) but has no access to language information which would allow it to represent its goals in terms of a language instruction. Object representations thus allow for a moderate level of abstraction in which geometrical information is preserved. They additionally open up the possibility for the agent of learning to achieve object-configuration goals irrespective of its particular point of view on the objects (invariance to geometrical similarity), which is highly desirable: a pyramid of blocks remains a pyramid regardless of the particular point of view taken by the builder on the scene. A final advantage of representations of goals and states as configurations of objects is their compositional nature (echoing the compositional nature of language): once the agent has learned to stack the blue block on the red block, if it generalizes well it should be able to stack the red block on the blue block.

For all the reasons outlined above, it is thus desirable to make a study of the ability of neural networks to learn to identify and discriminate configurations of objects. However, to our knowledge there is currently no controlled dataset or benchmark allowing to systematically study the problems outlined above. In this work, we introduce SpatialSim (Spatial Similarity), a novel spatial reasoning benchmark, to provide a well-defined and controlled evaluation of the abilities of models to successfully recognize and compare spatial configurations of objects. We divide this into two sub-tasks of increasing complexity: the first is called Identification, and requires to learn to identify a particular arrangement of objects; the second is called Discrimination, and requires to learn to judge whether two presented configurations of objects are the same, up to a change in point of view. Furthermore, we test and analyse the performance of increasingly connected GNNs in this task. We find that GNNs that operate on fully-connected underlying graphs of the objects perform better compared to a less-connected counterpart we call Recurrent Deep Set (RDS), to regular Deep Sets and to unstructured MLPs, suggesting that relational computation between objects is instrumental in solving the SpatialSim tasks. To summarize the key contributions of the paper:

- We introduce and motivate SpatialSim, a set of two spatial reasoning tasks and their associated datasets.
- We compare and analyze the performance of state of the art GNN models on these two tasks. We demonstrate that relational computation is an important component of achieving good performance.
- We provide preliminary analysis in the limits of these models in completely solving the benchmark.

## 2. The SpatialSim Benchmark

### 2.1. Description

**Figure 1:**
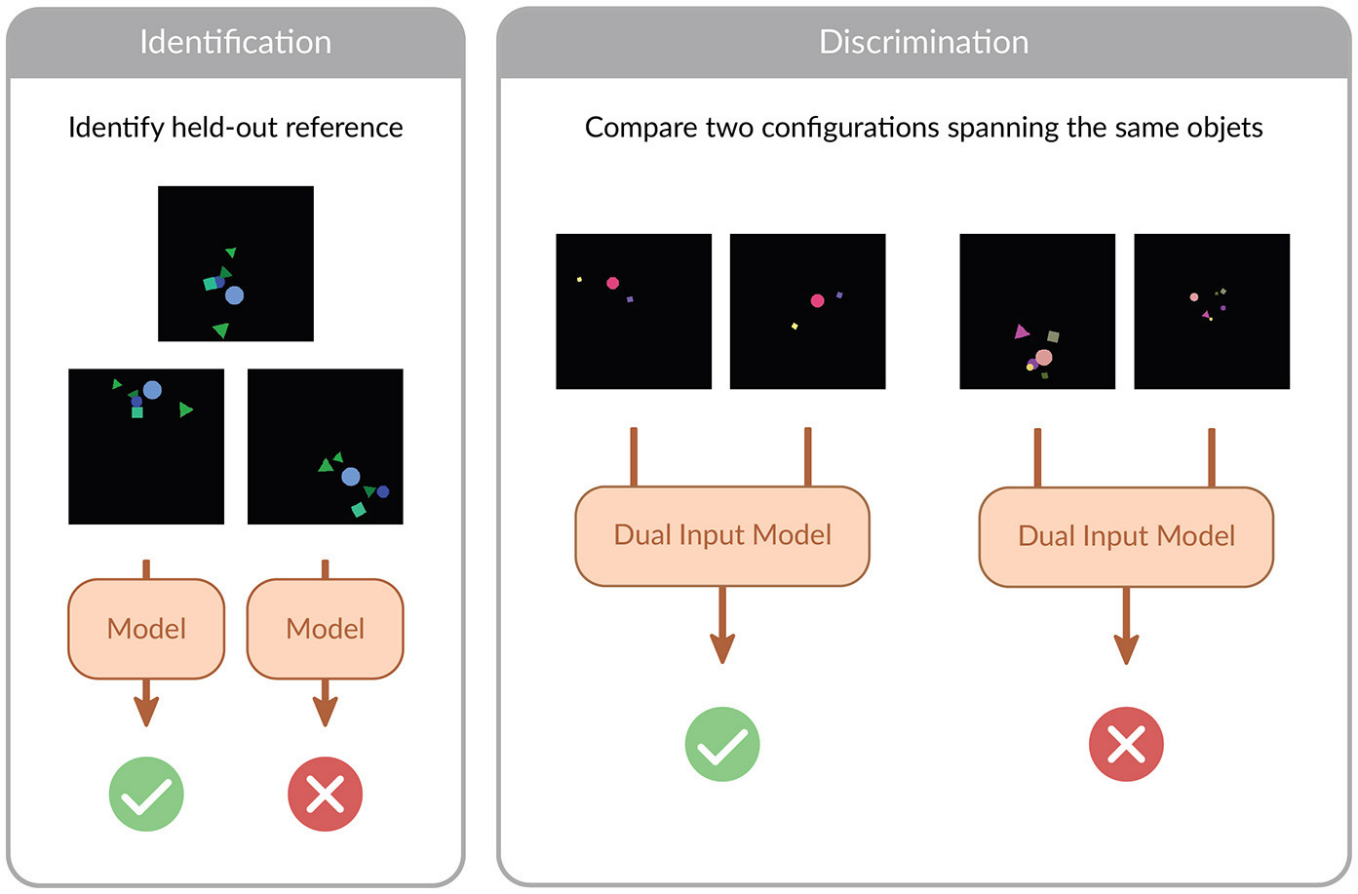
Visual illustration of SpatialSim. The benchmark is composed of two tasks, Identification and Discrimination. In Identification, the model is tasked with predicting whether a given configuration is the same as a held-out reference one, up to rotation, translation and scaling. In Discrimination, a dual-input model is tasked with recognizing whether a given pair of configurations is the same up to rotation, translation and scaling. We represent visual renderings of our objects, but note that for all objet-based models we consider the object-based representation is used as input: see main text.

In this work, we consider the problem of learning to recognize whether one spatial configuration of objects is equivalent to another. The notion of equivalence that we consider is grounded in the motivation outlined above: train models that can reason on configurations of objects regardless of their point of view. Because of that, we define equivalence in SpatialSim as geometrical similarity, e.g., any arbitrary composition of translations, rotations, and scalings. In general terms, we frame the problem as a classification problem, where positive examples are drawn from the same similarity equivalence class, and negative examples are drawn from a different one. Since we want, for this first study, to provide the simplest possible version of the problem, we place ourselves in the 2d plane. Extending the setting to 3 dimensions does bring some additional complexity but does not change the underlying mathematical problem (rotations in 2d can be parametrized by their origin and angle, so 3 dimensions, whereas rotations in 3d can be parametrized by their origin and euler angles, so 6 dimensions). We provide a visual illustration of the setting in Figure 1.

To provide a clean and controlled benchmark to study the ability of models to learn spatial similarity functions, we work with object features that are already given to the model. We ask the question: supposing we have a perfect feature extractor for objects in a scene, are we able to learn to reliably recognize spatial configurations? Working with objects directly allows us to disentangle the feature extraction process from the spatial reasoning itself.

We define a set of *n*_*obj*_ objects as colored shapes in 2d space, each uniquely characterized by a 10-dimensional feature vector. There are three possible shape categories, corresponding to squares, circles and triangles; the shape of each object is encoded as a one-hot vector. The shapes are distributed in continuous 2d space, and their colors belong to the RGB color space, where colors are encoded as a floating-point number between 0 and 1. The objects additionally have a size and an orientation, the latter given in radians. The feature vector for each object is the concatenation of all the previously described features, and a scene containing several objects is given as a set of the individual feature vectors describing each object. Note that the objects are provided without any order, and any permutation of the objects is possible encoding for a given scene; this is to test the permutation-invariance of the models. a highly desirable property.

We subdivide SpatialSim in two tasks. The first one, Identification, allows us to evaluate the abilities of different models to accurately summarize all relevant information to correctly respond to the classification problem. In this task, we sample a random configuration that will be the one the model in required to learn to identify. One configuration corresponds to one datasets, and we evaluate the capacities of the models on a set of configurations. The second task, Discrimination, allows us, in addition to the study allowed by the Identification task, to judge whether the computation learned by a model can be trained to be universal across configurations. For this purpose, the task requires to predict whether two distinct presented configurations belong to the same class or not. We give additional details on data generation for those two settings in the following sections, and a summary table in the Supplementary Material section A.

### 2.2. First Task: Identification

The Identification task is composed of several reference configurations of *n*_*obj*_ objects, each corresponding to a distinct dataset. Each sample of one dataset is either in the same similarity equivalence class as the reference configuration, in which case it is a positive example; or in a different similarity class as the reference, in which case it is a negative example. The same objects are present in all samples, not to give the model any additional information unrelated to spatial configurations.

This simple task allows us to isolate how well the model is able to learn as a function of the number of objects *n*_*obj*_: indeed, the model must make a decision that depends on the relationship between each objects, and for this purpose has to aggregate incoming information from *n*_*obj*_ vectors: when *n*_*obj*_ gets large the information the model is required to summarize increases. This can lead to loss of performance is the capacity of the models stays constant. For this reason, we structure the task with an increasing number of objects: we generate 27 datasets, with *n*_*obj*_∈[3..30]. We further group them into 3 collections of low number (*n*_*obj*_∈[3..8]), medium number (*n*_*obj*_∈[9..20]) and high number (*n*_*obj*_∈[21..30]).

For a given number of objects *n*_*obj*_ and based on one reference configuration generated by sampling *n*_*obj*_ random objects uniformly in 2d space, we generate a balanced dataset composed of:

**Positive examples**: After applying a small perturbation of factor ε, to each of the objects in the reference, very slightly changing their color, position, size and orientation, we apply a rotation around the configuration barycenter *B* with an angle ϕ~U([0,2π]), a scaling,of center *B* and magnitude s~U([0.5,2]), and a translation of vector *t*. The latter is sampled from the same distribution as the positions of the different objects.

**Negative examples**: These examples are generated by applying a small perturbation to the features of the objects in the reference, slightly changing their colors and sizes, and then re-sampling randomly the positions of the objects, while keeping the object's identity (shape, size, color). After randomly resampling the objects' positions, we apply a rotation, scaling and translation drawn from the same distribution as in the positive example to all objects. This is done to ensure no spurious correlations exist that could help models identify positive from negative examples regardless of actual information about the configuration. While it is, in theory, possible to sample a negative example that is close to the positive class, the probability is very low in practice for numbers of objects *n*≥ 3.

For each reference configuration, we generate 10,000 samples for the training dataset, and 5,000 samples, from the same distribution, for the validation and test datasets. For each dataset, we train a model on the train set and test it on the test set. The obtained test accuracies of the models over all datasets are averaged over a group (low, medium and high number of objects).

### 2.3. Second Task: Discrimination

In this section, we describe our second task. While in the previous setup the model had to learn to identify a precise configuration, and could learn to perform computation that does not generalize across configurations, we envision Discrimination as a more complex and complete setting where the model has to learn to *compare* two different configurations that are re-drawn for each sample. This task, while being more difficult than Identification, is also more general and more realistic: while sometimes an agent may be confronted with numerous repetitions of the same configuration that it has to learn to recognize—for instance, humans become, by extensive exposition, quite proficient at the task of recognizing the special configuration of visual elements that is human faces—but a very common task an intelligent agent will be confronted to is entering a new room filled with objects it knows but that are arranged in a novel way, and having to reason on this precise configuration.

For this task, because each sample presents a different set of objects, *n*_*obj*_ can vary from one sample to the other, and thus a single dataset can cover a range of number of objects. We generate three distinct datasets, one with *n*_*obj*_∈[3..8], one with *n*_*obj*_∈[9..20] and one with *n*_*obj*_∈[21..30]. In preliminary experiments we have observed learning the Discrimination task is very hard, leading to a great dependence on the initialization of networks: some seeds converge to a good accuracy, some don't perform above chance. This is due to the presence of rotations in the allowed transformations in the positive examples; a dataset containing only translations and scalings leads to good learning across initializations. Note that this problem with rotations persists for a simplified setup containing only configurations of *n*_*obj*_ = 3 objects. To alleviate this and carry the optimization process we introduce a curriculum of five datasets, each one with a different range of allowed rotations in the generation process, with the last one spanning all possible rotation angles.

We generate the dataset as:

**Positive examples**: We draw the first configuration by randomly sampling the objects' shape, size, color, position and orientation. For obtaining the second configuration, we copy the first one, apply a small perturbation to the features of each object, and apply a random rotation, scaling, and translation to all objects, using the same process as described in the Identification task.

**Negative examples**: We draw the first configuration as above, apply a small perturbation of magnitude ε and for the second one we randomly re-sample the positions of each object independently, while keeping the other features constant. We finally apply a random rotation, scaling and translation and this gives us our second set of objects.

For each range of number of objects and for each dataset of the curriculum we generate 100,000 samples for the training set. We generate a validation and a testing set of 10,000 samples for each range of *n*_*obj*_. Those datasets contain rotations in the full range.

## 3. Models and Architectures

With the benchmark, we establish a first set of reference results from existing models in the in the literature, serving to identify their strengths and limits, and as baselines for further work. We consider Message-Passing GNNs for their established performance, notably in physical reasoning tasks, along with stripped-down versions of the same models. Additionally, our hypothesis is that models that implement relational computation between objects will perform best in this benchmark, because it requires taking into account the relative positions between objects and not only their absolute positions in 2d-space. To test this hypothesis, we model a configuration of objects as a graph, where the individual objects are the nodes. We then train three GNN models with decreasing levels of intra-node communication: MPGNN (full Message-Passing GNN, see Gilmer et al., 2017) performs message-passing updates over the complete graph where all object-object edges are considered; GCN (Kipf and Welling, 2016) computes updated node representations based on the sun-aggregation of linearly transformed node representations from incoming edges, RDS (Recurrent Deep Set) is a Deep Set model (Zaheer et al., 2017) where each object updates its features based on its own features and a global vector aggregating all the other object features (all-to-one message passing); and a regular Deep Set model where each node updates its own features independently.

We additionally compare, for both tasks, our models to Multi-Layer Perceptron (MLP) baselines. Our MLP baselines are built to have the same order of number of hidden units as the GNNs, to allow for similar representational capacity. However, because there is a considerable amount of weight sharing in GNNs compared to MLPs the number of weights is much higher, and additionally, increases substantially with the number of objects. More details on the MLP baselines are given in the Supplementary Material section C. As a final baseline, and to validate our claims on the importance of relational inductive biases on geometrical reasoning, we compare those architectures operating on object-based input with ResNet18-based architectures operating directly on pixel input.

For all GNN models, after *N* node updates, the node features are summed, passed through an MLP to output a two-dimensional vector representing the score for the positive and negative class. For the Discrimination task, the models take as input two configurations, pass them through two parallel GNN layers, concatenate their output embeddings and pass this vector through a final MLP to produce scores for both classes. For a detailed description of all models, including a visual overview, please refer to the Supplementary Material section C.

## 4. Experimental Results

### 4.1. Identification

In this section we report the experimental results for the Identification task. Reported results are averaged over 10 independent runs and across datasets in the same group (low, medium, high number of objects). For a fair Discrimination across parameter numbers in the GNNS, we build each internal MLP used in the message computation, node-wise aggregation, graph-wise aggregation and prediction steps with the same number of hidden layers *d* and the same number of hidden units *h*. Since the DS and RDS layer can be seen as stripped-down versions of the MPGNN layer, if *d* and *h* stay constant the number of parameters drops for the RDS layer, and drops even further for the DS layer. To allow for a fair Discrimination with roughly the same number of parameters in each model, we use *h* = 16 for all architectures and *d* = 1 for the MPGNN, *d* = 2 for the RDS and *d* = 4 for the DS, and we report the number of parameters in the table. For the embedding vector that aggregates the whole configuration information, we use a dimension *h*_*u*_ = 16: thus the number of objects is at first small compared to *h*_*u*_ and gradually becomes larger as *n*_*obj*_ increases. We found no significant difference between using one or several rounds of node updating in the GNNs, so all our results were obtained with *N* = 1. We train for 20 epochs with the Adam optimizer (Kingma and Ba, 2014), with a learning rate of 10^−3^ and a batch size of 128.

We report the means and standard deviations of the test accuracies across all independent runs in Table 1, as well as the numbers of parameters for each model. Chance is at 0.5. For object-based models, we observe the highest accuracy with the MPGNN model, on all three object ranges considered. It achieves upwards of 0.97 percent accuracy, effectively solving the task for numbers of objects ranging from 3 to 30. Note that in this range performance of the MPGNN stays constant when the number of objects increases. In contrast, the RDS model achieves good performance (0.91) when the number of objects is low, but its performance decreases as the number of objects grows. GCNs perform barely above chance in this task, possibly due to the lower expressivity of linear layers compared to MLPs in the relation operation. Deep Sets show lower performance in all cases, and the MLP achieves 0.85 mean accuracy with *n*_*obj*_∈[3..8] but its performance drops sharply as *n*_*obj*_ increases. Finally, we can see that the CNN-based model completely solves the task from pixel input, no matter the number of objects. This suggests that solving this task is possible by simply pattern-recognizing the positive examples with a high-capacity model.

**Table 1:** Test classification accuracies (means and standard deviations are given over datasets and seeds) for the three different models on the Identification task.

**Model**	***n*_*obj*_∈[3..8]**	***n*_*obj*_∈[9..20]**	***n*_*obj*_∈[21..30]**	**Parameters**
MPGNN	0.97± 0.026	0.98 ± 0.024	0.98 ± 0.028	2,208
GCN	0.54 ± 0.033	0.52 ± 0.014	0.51 ± 0.013	2530
RDS	0.91 ± 0.062	0.85 ± 0.128	0.78 ± 0.19	2,038
Deep Set	0.65 ± 0.079	0.60 ± 0.082	0.58 ± 0.09	2,386
ResNet18	**0.99** ± 0.017	**1.0** ± 0.007	**1.0** ± 0.013	11.7M
MLP Baseline	0.82 ± 0.09	0.59 ± 0.051	0.56 ± 0.051	6k/48k/139k

*Bold values indicate highest average accuracy*.

Our results provide evidence for the fact that, while it is possible to identify a given configuration with well-above chance accuracy without performing any relational computation between objects, to effectively solve the task it seems necessary to perform fully-connected message-passing between the objects, especially when the number of objects increases. However, while the number of parameters stays constant in a fully-connected MPGNN when *n*_*obj*_ increases, the amount of computation scales as the number of edges [$O(n_{obj}^{2})$]. This makes using MPGNNs on complete graphs harder to use at scale. However, note that we consider this task as an abstraction for naturally-occurring configurations of objects that contain a limited number of objects, so this quadratic increase in time complexity should not be a problem in practice. Additionally, we provide evidence that this task is efficiently solvable by large, unstructured architectures.

### 4.2. Discrimination

We now turn to our Discrimination task. We compare the dual-input architecture with different internal layers: MPGNN, GCN, RDS and DS, and to MLP and ResNet18 baselines. The MLP is built according to the principle stated above, and takes as input a concatenation of all the objects in both configurations. As in the previous section, to ensure a fair comparison between models in terms of the number of parameters we provide our GCN, RDS and DS layers with deeper MLPs (see section C on model details in Supplementary Material). We train models on three datasets, respectively with *n*_*obj*_∈[3..8], *n*_*obj*_∈[9..20] and *n*_*obj*_∈[21..30], for 10 seeds per dataset, in Table 2. For this task, we train models with the Adam optimizer on 5 epochs on each curriculum dataset (25 epochs total) with a batch size of 128 and a learning rate of 10^−3^.

**Table 2:** Test classification accuracies for the three different models on the discrimination task.

**Layer type**	***n*_*obj*_∈[3..8]**	***n*_*obj*_∈[9..20]**	***n*_*obj*_∈[21..30]**	**Parameters**
MPGNN	**0.89** ± 0.030	**0.81** ± 0.121	**0.71** ± 0.176	4,686
GCN	0.55 ± 0.006	0.50 ± 0.004	0.50 ± 0.05	4,962
RDS	0.8 ± 0.133	0.68 ± 0.154	0.52 ± 0.04	5,326
Deep Set	0.51 ± 0.014	0.50 ± 0.001	0.50 ± 0.005	5,274
ResNet18	0.50 ± 0.002	0.50 ± 0.004	0.50 ± 0.005	11.7M
MLP Baseline	0.55 ± 0.002	0.51 ± 0.006	0.50 ± 0.004	26k/192k/552k

*All metrics were computed on 10 different seeds and trained for 5 epochs on each dataset of the curriculum. Bold values indicate highest average accuracy*.

We report mean accuracies of the different models and their standard deviation in Table 2. As before, chance performance is 0.5. We immediately see the increased difficulty of Discrimination compared to Identification: the model based on MPGNN layers performs best, with mean accuracies of 0.89, 0.81 and 0.71, compared to a performance above 0.97 on Identification. In this case we see the performance drop for MPGNN when *n*_*obj*_ increases. RDS performs well above chance when the number of objects is small, but its performance drops rapidly afterwards. GCN performs slightly above chance in the condition with low number of objects, but its performance drops to chance levels afterwards. Both Deeps Sets and the MLP fail to reliably perform above chance. Interestingly, while ResNet18-based models were able to completely solve Identification, they fail to perform above chance in Discrimination, highlighting the harder nature of the task. While ResNet's high capacity allowed it to memorize the positive examples in Identification, the dynamic nature of Discrimination (new configurations are presented at each sample) creates difficulty for unstructured models. As concerns object-based models, we established in the previous section that complete message-passing between nodes is instrumental in learning to identify particular configurations. The experiments in the Discrimination task suggest that layers that allow nodes to have access to information about other nodes are key to achieve good performance, whether this communication is centralized, via conditioning by the graph-level feature as in the RDS, or decentralized, as allowed by the MPGNN layer. Additionally, full node-to-node communication seems to be crucial for good performance, and we show in the next subsection how this affects the functions learned by the models. However, it does not seem to be enough to completely solve the task. We provide additional details on the generalization properties of the models in the Supplementary Material sections E–G.

### 4.3. Model Heat Maps

**Figure 2:**
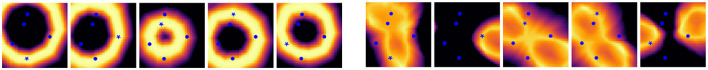
Magnitude of the difference in predicted score for the positive and negative classes for a comparison between a 5-object configuration and a perturbed version of this configuration where one object is displaced over the 2d-plane. Value displayed is *score*_+_−*score*_−_, where *score*_+_ corresponds to the score of the logits of the positive class as output by the model, and *score*___ corresponds to the logits of the negative class. Left row is with RDS layers and right row is with MPGNN. For each row, the displaced object's position is indicated with a blue star, the other ones with a blue dot. The sizes, colors, orientations and shapes of the objects are not represented. Bright yellow means the model assigns the positive class to the configuration where the displaced object would be placed here, black means the negative class would be assigned.

In addition to the above quantitative results, we assess the quality of the learned functions for different models. We do this by visualizing the magnitude of the difference between the scores of the positive and negative classes, as output by the different models, as a function of the position of one of the objects in the configuration. We show the results in Figure 2. Beyond the clear qualitative differences between different models, this figure clearly shows the shortcomings of the models. A perfect model for this task would show a high-magnitude region in the vicinity of the considered object, and low values everywhere else. Ideally, the object would be placed at a global maximum of this score difference function. Instead, both the RDS and MPGNN models show extended crests of high magnitudes: this means that moving the considered object along those crests would not change the prediction of the models, whereas the configurations are clearly changed. This suggests that all models we considered are limited in their capacity to distinguish classes of similar configurations. This lack of a clearly identifiable global maximum over variations of the position of one object suggests a possible reason for ceiling in performance exhibited by our models: the tested GNNs are unable to break certain symmetries. For the RDS, since each node only has access to global information about an aggregate of the other nodes, it is not surprising to see it exhibit the radial symmetry around the barycenter of the configuration. MPGNNs seem to operate in a different way: for each node the learned function seems to show symmetry around axes related to the principal directions of the distribution of other nodes. The models are thus unable to discriminate a large subspace of the configuration space (e.g., the RDS is insensitive to the rotation of one object around the barycenter of the configuration, since the object stays in the high-magnitude zone). More discussion on this subject can be found in the Supplementary Material section D.

### 4.4. Generalization to Different Numbers of Objects

We have highlighted in the previous section some qualitative measures of the shortcoming of our models in the proposed tasks. To complete these results, in this section we present a generalization experiment for the Discrimination task. Since the models for this task are trained on any couple of configurations, they can be transferred to datasets with higher numbers of objects. In this experiment we train Deep Set, RDS and MPGNN models on one dataset (*n*_*obj*_∈[3..8], *n*_*obj*_∈[9..20], or *n*_*obj*_∈[21..30]) and test the models on all three datasets. The results are reported in Table 3.

**Table 3:** Generalization results between datasets for Deep Set, RDS and MPGNN. The numbers plotted are averages of testing accuracies.

	**3–8**	**9–20**	**21–30**
3–8	0.51 ± 0.016	0.49 ± 0.046	0.50 ± 0.043
	0.80 ± 0.133	0.66 ± 0.138	0.51 ± 0.048
	**0.89** ± 0.03	**0.71** ± 0.092	**0.56** ± 0.075
9–20	0.51 ± 0.046	0.50 ± 0.001	0.50 ± 0.047
	**0.75** ± 0.125	0.68 ± 0.154	0.52 ± 0.054
	0.68 ± 0.063	**0.81** ± 0.121	**0.68** ± 0.16
21–30	0.50 ± 0.04	0.51 ± 0.068	0.50 ± 0.05
	**0.60** ± 0.087	0.68 ± 0.15	0.52 ± 0.04
	0.51 ± 0.048	**0.77** ± 0.12	**0.71** ± 0.18

*Columns correspond to training datasets, rows to testing datasets. Each block corresponds to one train-set/test-set combination. In each block, the results are given from top to bottom for Deep Set, RDS and MPGNN. Diagonal blocks correspond to matching train set/test set combinations. All reported results are averages and standard deviations over 10 different runs. Rows and columns are annotated with the n_obj_ range. Bold values indicate highest average accuracy*.

The results demonstrate the limited abilities of the models to transfer their learned functions to higher or lower numbers of objects. For instance, MPGNNs achieve 0.89 test accuracy when trained *and* tested on 3–8 objects, but this performance decreases sharply on the datasets with higher numbers of objects. This is less the case for RDS, presumably because the simpler functions they learn, while achieving lower performance when tested on the matching dataset, are more robust to higher numbers of objects. Another interesting point is that models trained on 9 to 20 numbers of objects appear to transfer better than other conditions. In particular, both RDS and MPGNN achieve higher mean test accuracy when transferring from 9–20 objects to 21–30 objects than models which were directly trained on 21–30 numbers of objects. The 21–30 dataset is harder to train on, so the models trained directly on this dataset may never learn, which bring the mean accuracy down. This suggests that functions useful for good performance on 9–20 numbers of objects are also useful for 21–30 numbers of objects. In contrast, functions useful for good performance on 3–8 numbers of objects do not transfer well to higher numbers of objects.

This evaluation suggests a tradeoff in being able to solve the task well for low numbers of objects vs. being able to solve the task for high numbers of objects. This confirms the qualitative evaluation in Section 4.3, where we remarked that the functions learned by the models varied greatly with the dataset they were trained on.

## 5. Related Work

### 5.1. Spatial Relations and Language

While previous work in Visual Question Answering (VQA Antol et al., 2015) and Instruction-Following Reinforcement Learning (Luketina et al., 2019) have considered issues related to the ones we consider in SpatialSim, such work is constrained to using spatial relations that can be labeled by natural language. For instance, one of the standard benchmarks in VQA is the CLEVR dataset (Johnson et al., 2016). In CLEVR, questions are asked about an image containing a collection of shapes. The questions themselves are more designed for their compositionality (“the object that is of the same color as the big ball that is left of …”) than for geometric reasoning *per se*. While the dataset does contain questions related to spatial reasoning, there are very few of them (four) and they are of a different nature than the precise reasoning on configurations that we wish to address. For instance, the concepts of “left of” or “right of,” while defining broad spatial relations, are not invariant to the point of view of the observer. Since language abstracts away geometry, such a benchmark is unsuitable to the study of geometrical reasoning. Consider the task of stacking rocks of different shapes to make a tower; a language description could be “stack this rock on top of this one which is on top of this one etc …” But the “on top of” does not capture the precise positional information that allows a rock-stacking agent to estimate the centers of mass to correctly balance its construction. Their linguistic nature makes those datasets unsuitable for investigating the question of learning to identify and compare precise geometrical arrangements of objects. A follow-up to CLEVR is the CLEVRER dataset and tasks (Yi et al., 2020). While this benchmark is very thorough, requiring solutions to be able to perform prediction as well as counterfactual reasoning, we would argue that the tested abilities are a bit broad. In this work, we focus on a more restrained task that allows us to test the abilities of our models to perform recognition of equivalence classes of objects.

### 5.2. Graph Neural Networks

This is based on the recent line of work on neural networks that operate on graph-structured input. Seminal work (Gori et al., 2005; Scarselli et al., 2009) involved updating the representations of nodes using a contraction map of their local neighborhood until convergence, a computationally expensive process. Follow-up work alleviated this, by proposing neural network models where each layer performs an update of node features based on the previous features and the graph's adjacency matrix, and several such layers are stacked to produce the final output. Notably, Graph Convolutional Networks (GCNs) (Bruna et al., 2014; Defferrard et al., 2016; Kipf and Welling, 2016), in their layers, use a linear, first-order approximation of spectral graph convolution and proved effective in several domains (Duvenaud et al., 2015). However, these works have focused on working on large graphs where the prediction at hand depends on its connectivity structure, and the GCN can learn to encode the structure of their k-hop neighborhoods (for k computation steps). In our case there are a lot less objects, and the precise connectivity is irrelevant since we consider GNNs on fully-connected graphs.

A different class of networks (MPGNNs) has been proposed to more explicitly model an algorithm of message-passing between the nodes of a graph, using the edges of the graph as the underlying structure on which to propagate information. This is the class of model that we consider in this work, because of their flexibility and generality. A variant of this was first proposed to model objects and their interactions (Battaglia et al., 2016; Santoro et al., 2017) by explicitly performing neural computation on pairs of objects. The full message-passing framework was introduced in Gilmer et al. (2017) for supervised learning on a molecular dataset, and was expanded in Battaglia et al. (2018), which provided a unifying view of existing approaches.

A line of theoretical work has focused on giving guarantees on the representational power of GNNs. Xu et al. (2018) introduced a simple model that is provably more powerful than GCNs, and universality of approximation theorems for permutation invariant (Maron et al., 2019) and permutation equivariant (Keriven and Peyré, 2019) functions of nodes of a graph have also derived recently. However, the models constructed in these proofs are theoretical and cannot be tractably implemented, so this line of work opens interesting avenues for empirical research in the capacities of GNNs in different practical settings. In particular, Xu et al. (2018) found that GNNs have powerful theoretical properties if the message-aggregation function is injective. This means that the aggregation function has to preserve all the information from its input, which is a collection (of a priori unbounded size) of vectors, to its output, which is a single vector of bounded dimension. Our setting allows us to examine the ability of GNNs to appropriately aggregate information in an experimental setting.

Finally, our work relates to recent work on learning distance functions over pairs of graphs (Ma et al., 2019). Recent work (Li et al., 2019), in a model they call Graph Matching Network, has proposed using a cross-graph node-to-node attention approach for solving the problem, and compared it to an approach without cross-graph attention, that is closely related to our models for the Discrimination task. However, our work here is distinct, not so much in the actual architectures used, but in the properties of the setting. Their work considered a task related to graph isomorphism, where the precise connectivity of the graph is important. In this work we are interested in the features of the nodes themselves and we consider the underlying graph simply as a structure supporting the GNN computation.

### 5.3. Learning Same-Difference Relations in Convolutional Neural Networks

Related to our work in a recent line of contributions looking to examine the relational reasoning capacities of machine vision systems, in particular of Deep Convolutional Neural Networks (DCNNs) (see Ricci et al., 2021 for a recent review). This line of work focuses on tasks where a network has to classify images containing two similar or different shapes, usually the two similar shapes being translated and scaled versions of each other. This task, often studied in humans (since Shepard and Metzler, 1971 in the case of mental rotations), entails abstract relational visual capabilities and it has been shown to be solved by non-human primates (Donderi and Zelnicker, 1969), birds (Katz and Wright, 2006), rodents (Wasserman et al., 2012), and insects (Giurfa et al., 2001). Kim et al. (2018) have shown that regular DCNNs and Relation Networks (Santoro et al., 2017) have difficulties learning the same-different abstract categories, a problem that is alleviated by using a Siamese architecture taking as input each of the shapes, a process the authors link to perceptual grouping of objects in humans. Relatedly, Puebla and Bowers (2021) have shown that DCNNs evaluated on the same-different task are not robust to changes in the low-level features of the underlying images, arguing that representations of objects and their relations are needed. This is the setting in which we place ourselves in our second task, Discrimination, where object representations are directly accessible to networks with varying amounts of relational computation. In addition, we consider rotational invariance as part of our equivalence classes, which has not been considered in this line of work.

### 5.4. Unsupervised Object Discovery Across Time

In recent years there also has been a trend toward using object-centric approaches to physical reasoning, by learning unsupervised latent representations of objects from videos of dynamic object interaction. Constrastive Structured World Models (C-SWMs) (Kipf et al., 2019) learn, from pixel input, a latent representation in as a set of entities, after having passed an encoder; the latents are trained in a contrastive fashion, with positive pairs being continuous in time and negative pairs being randomly matched. This contrastive training scheme ensures the latents capture meaningful information without having to compute any loss in pixel space. AlignNet and OAT (Creswell et al., 2020, 2021) also operate on raw videos of dynamic objects, using a MONet encoder (Burgess et al., 2019) to produce a latent representation of a set of objects. The goal of this work is to find, for any given time step, a permutation between object indices such as the objects resulting from the encoding are always enumerated in the same order. As the focus of the work is not object interaction, their dynamics model is not relational, and is based on an LSTM acting on entity features. The OP3 model (Veerapaneni et al., 2020) builds upon a similar idea than IODINE (Greff et al., 2020)—that is, inferring the entities present in a scene through iterative refinement—extended to temporally-extended image inputs; as such, it also includes a dynamics model based on a GNN. The authors show that using their approach in model-based planning leads to better generalization compared to using unstructured or non-relational approaches. These models have in common that the entity encoder is separate from the dynamics model which operates over a structured latent space. While physical reasoning is feasible using non-relational models (Eslami et al., 2018), each of these works provide evidence for the usefulness of object-centric inductive biases as well as of the separation between object perception models and physics models. SpatialSim focuses on the object-relation component, but this previous line of work suggests how well-performing object encoders could be extracted, even with a very simple encoding scheme (as in the case in C-SWMs), from the spatial and temporal structure of observations of interacting objects. However, the implicit spatial reasoning tasks that are solved in these tasks are somewhat easier than what is needed in the SpatialSim benchmark because the worlds they consider have smooth transitions: contiguous frames are close to each other in pixel and entity space, which is not the case in our setup.

## 6. Conclusion

In this work, we motivated the use of GNNs to learn goal-achievement functions over equivalences classes of spatial configurations of objects. We introduced SpatialSim, a simplified but challenging spatial reasoning benchmark that serves as a first step toward more general geometrical reasoning where a model has to learn to recognize an arrangement of objects irrespective of its point of view. We demonstrated that the relational inductive biases exhibited by Message-Passing GNNs is crucial in achieving good performance on the task, compared to a centralized message-passing scheme or to independent updating of the object features. MPGNNs achieve near-perfect performance on the Identification task, but achieve much lower performance on the Discrimination task. Our experiments with ResNets suggest that object-centered architectures are also instrumental in solving the difficult Discrimination task, as demonstrated by the failure of CNNs with 4 order of magnitude more parameters than the small relational neural networks we consider. Our analysis suggests two shortcomings of current models on this benchmark: 1) the models struggle to accurately summarize information when the ratio between the number of objects and the size of the embedding used for representing the whole configuration becomes large and 2) GNNs in spite of their relational inductive biases struggle to break certain symmetries; we take this to mean additional theoretical and experimental research is needed to find more appropriate biases for geometrical reasoning.

## Data Availability Statement

The datasets presented in this study can be found in online repositories. The names of the repository/repositories and accession number(s) can be found below: https://github.com/laetitia-teo/spatial-sim.

## Author Contributions

LT: performed experiments and wrote the manuscript. P-YO and KH: supervised and wrote the manuscript. All authors contributed to the article and approved the submitted version.

## Conflict of Interest

KH was employed by the company Microsoft. The remaining authors declare that the research was conducted in the absence of any commercial or financial relationships that could be construed as a potential conflict of interest.

## Publisher's Note

All claims expressed in this article are solely those of the authors and do not necessarily represent those of their affiliated organizations, or those of the publisher, the editors and the reviewers. Any product that may be evaluated in this article, or claim that may be made by its manufacturer, is not guaranteed or endorsed by the publisher.
