# Supplementary material for: SpatialSim: Recognizing Spatial Configurations of Objects With Graph Neural Networks
Source: Front Artif Intell. 2022 Jan 26;4:782081. doi: 10.3389/frai.2021.782081 (PMC8826049; doi:10.3389/frai.2021.782081)
Supplement: Supplementary file 1 [file Data_Sheet_1.PDF]

Supplementary Material

SUPPLEMENTARY MATERIAL: SUMMARY

This document provides additional details on the SpatialSim benchmark, the architectures and models used, and some additional experimental results and analysis. It is organized in the following way:

- **Section a: SpatialSim Benchmark Summary**
- **Section b: Additional Details on Dataset Generation**
- **Section c: Models and Architectures**
- **Section d: Model Heatmaps; Additional Discussion**
- **Section e: Easier and Harder Configurations**
- **Section f: Generalization over Object Number**
- **Section g: Effects of Variations in Number of Training Examples**
- **Section h: Adding Distractor Objects**

A SPATIALSIM BENCHMARK SUMMARY

This section provides a summary of the SpatialSim benchmark.

The datasets, as well as the code and instructions to reproduce our experiments, are accessible at the following link: https://sites.google.com/view/gnn-spatial-reco/. We also provide the dataset generation code to produce extended versions of our datasets.

All datasets belonging to both SpatialSim tasks are detailed in Table a.

As described in the main text, the Discrimination task is harder to train on than the Identification task. This is because of the presence of rotations in the allowed transformation for the same similarity class. This problem does not show when rotations are not included in the dataset. To help the optimization process, we generate a curriculum of datasets with a set of increasing ranges for allowed rotation angles θ, up to the entire $[0, 2\pi]$ range. We thus generate, for each n_{obj} condition (*low*, *mid*, *high*) a set of 5 datasets with respective7 allowed rotation angles θ:

- $\theta \in [0, \frac{\pi}{10}]$
- $\theta \in [0, \frac{\pi}{2} + \frac{\pi}{10}]$
- $\theta \in [0, \pi + \frac{\pi}{10}]$
- $\theta \in [0, \frac{3\pi}{2} + \frac{\pi}{10}]$
- $\theta \in [0, 2\pi]$

For each condition the test set is unique and has $\theta \in [0, 2\pi]$: we test on unrestrained rotations. This curriculum is used with all our models in all our experiments.

Names of the datasets: the datasets presented in Table a are named in the following way.

- **For Identification**, the 'IDS' prefix is followed by n_{obj} and then by the '_valid' and '_test' suffix respectively for validation and test sets.

	Identification		Discrimination	
Condition<br>*low* $n_{obj} \in$<br>[3..8]	IDS_3<br>IDS_4<br>IDS_5<br>IDS_6<br>IDS_7<br>IDS_8	IDS_3_test<br>IDS_4_test<br>IDS_5_test<br>IDS_6_test<br>IDS_7_test<br>IDS_8_test	CDS_3_8_0<br>CDS_3_8_1<br>CDS_3_8_2<br>CDS_3_8_3<br>CDS_3_8_4	CDS_3_8_test
Condition<br>*mid* $n_{obj} \in$<br>[9..20]	IDS_9<br>IDS_10<br>IDS_11<br>IDS_12<br>IDS_13<br>IDS_14<br>IDS_15<br>IDS_16<br>IDS_17<br>IDS_18<br>IDS_19<br>IDS_20	IDS_9_test<br>IDS_10_test<br>IDS_11_test<br>IDS_12_test<br>IDS_13_test<br>IDS_14_test<br>IDS_15_test<br>IDS_16_test<br>IDS_17_test<br>IDS_18_test<br>IDS_19_test<br>IDS_20_test	CDS_9_20_0<br>CDS_9_20_1<br>CDS_9_20_2<br>CDS_9_20_3<br>CDS_9_20_4	CDS_9_20_test
Condition<br>*high* $n_{obj} \in$<br>[21..30]	IDS_21<br>IDS_22<br>IDS_23<br>IDS_24<br>IDS_25<br>IDS_26<br>IDS_27<br>IDS_28<br>IDS_29<br>IDS_30	IDS_21_test<br>IDS_22_test<br>IDS_23_test<br>IDS_24_test<br>IDS_25_test<br>IDS_26_test<br>IDS_27_test<br>IDS_28_test<br>IDS_29_test<br>IDS_30_test	CDS_21_30_0<br>CDS_21_30_1<br>CDS_21_30_2<br>CDS_21_30_3<br>CDS_21_30_4	CDS_21_30_test

Table S1. Summary Table for SpatialSim, listing all datasets. The two main columns correspond to the two tasks. The three main rows correspond to the three object number condition: low, mid, and high. For each task/object number condition combination, the different datasets are listed according to whether they are train or test datasets. Validation datasets are omitted from the table for clarity, but are drawn from the same distribution as the test sets, and are available at the provided link. Note that Identification has a dataset for each configuration (one per number of objects) and that Discrimination has five train dataset for each valid/test set corresponding to the curriculum in rotation angles described above.

- **For Discrimination**, the 'CDS' prefix is followed by the range of numbers of objects (the dataset may contain samples with n_{obj} in this range, inclusive). The training datasets additionaly have an identifier corresponding to their place in the rotation angle curriculum (0 to 4, in the above-defined order). The validation and test have the '_valid' and '_test' suffix, respectively.

B DATASET CREATION

In this section we give additional information on dataset creation. We consider the world as square with length and width 20 units. We sample the x and y positions of our objects in this square. The sizes of our objects describe their radius (an object of size s is contained in a square of side $2s$) and range from 0.5 to 2 units. For orientation, we used the following approximation: we considered orientation as a one-dimensional variable, expressed in radians, and we sample the objects' orientation between 0 and 2π. This is an approximation because the periodic nature of angles cannot be represented in one dimension. The colors of the objects are sampled in the continuous 3d RGB space, and each component ranges from 0 to 1. As for shapes, there are 3 possible categories (square, circle, triangle) that are represented by a corresponding one-hot vector.

C MODELS AND ARCHITECTURES

c.1 Models for Identification

In this section we present our graph creation procedure for the Identification task and provide the equations for the models we use: Message-Passing GNN, Recurrent Deep Set, Deep Set and MLP. We additionally present a visual illustration of our different layers in Figure S1.

c.1.1 Graph Creation

From a set of objects S we construct a fully-connected, directed graph G that is used as an input to our GNN. In our work, $G = (X, A, E, u)$ contains the following information :

- $X \in \mathbb{R}^{n \times d_x}$ is a tensor of node features, containing a vector of dimension d_x for each of the objects in the scene;
- $A \in \mathcal{M}^{n \times n}$ is the adjacency matrix of the graph;
- $E \in \mathbb{R}^{e \times d_e}$ is a tensor of edge features, also referred to as messages in the rest of this article, labeling each of the e edges with a d_e-dimensional vector, and that can be seen as information propagating from the sender node to the receiver node. We choose the dimensionality of edges to be twice the dimensionality of nodes d_x;
- u is a graph-level feature vector, used in the GNN computation to store information pertaining to the whole graph, and effectively used as an embedding of the graph to predict the class of the input.

Initialization of the graph : Since our models require inputs for E and u that are not *a priori* given in the description of the collection of objects, we use a generic initialization scheme : u is initialized with the mean of all node features, and each edge is initialized with the concatenation of the features of the sender node and the receiver node.

c.1.2 Message-Passing GNN

The MPGNN can be seen as a function operating on graph input and producing a graph output: GNN : $G(X, A, E, u) \to G'(X', A, E', u')$, where the dimensionnality of the node features, edge features and global features can be changed by the application of this function, but the graph structure itself encoded as the adjacency matrix A is left unchanged. This GNN can then be described as the composition of several functions, each updating a part of the information contained in the graph :

Message computation : We denote by $E_{i \to j}$ the feature vector of the edge departing from node i and arriving at node j, X_i the feature vector of node i, and $[x||y]$ the concatenation of vectors x and y, and by MLP a multi-layer perceptron. The message passing step is then defined as :

$$E'_{i \to j} \leftarrow MLP_E([X_i||X_j||E_{i \to j}||u])$$

At each time step, the message depends on the features of the sender and receiver nodes, the previous message, and the global vector u.

Node-wise aggregation : Once the message along each edge is computed, the model computes the new node features from all the incoming edges. We define by $\mathcal{N}(j)$ the incoming neighbourhood of node j, that is, the set of nodes $i \in [1..n]$ where there exists an edge going from i to j. The node computation is then performed as so :

$$X'_j \leftarrow MLP_X\left(\left[X_j\middle|\middle|\sum_{i \in \mathcal{N}(j)} E'_{i \to j}\middle|\middle|u\right]\right)$$

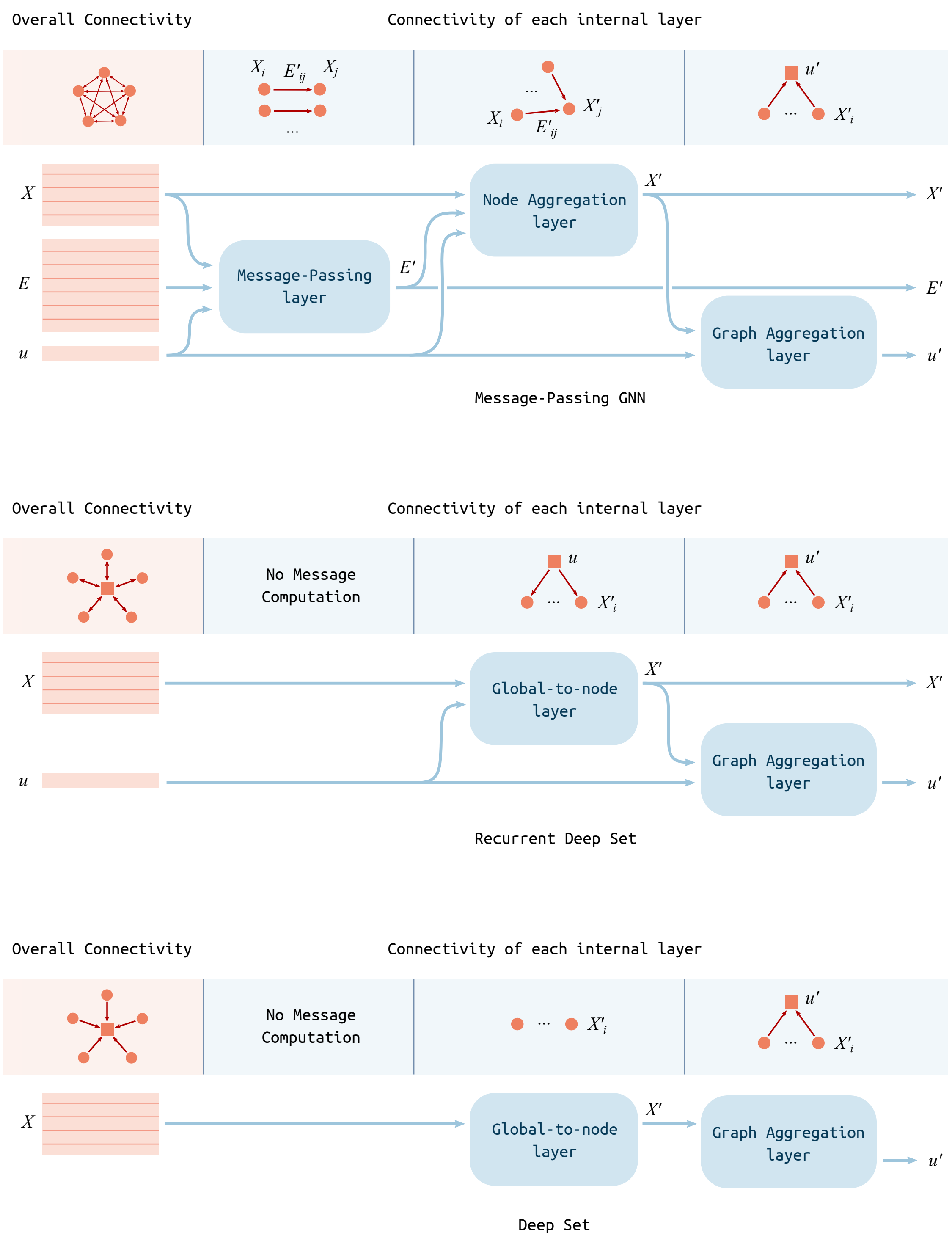

Figure S1. An illustration of the three different layers used in this work. Going from MPGNN to RDS to DS can be seen as an ablation study, where different elements are withdrawn from the layer to study their impact on final performance. For the MPGNN and RDS layers, the output tensors are then fed back as inputs of the model, providing recurrent computation; this is not the case for the Deep Set layer. In this figure, emphasis is put on the connectivity implied by each layer. Nodes are represented by orange disks, the graph-level embedding, which can be seen as a special kind of node, is represented with an orange square. From top to bottom, we go from all-to-all connectivity to bidirectional all-to-one to unidirectional all-to-one.

Graph-wise aggregation Finally, we update the graph-level feature, that we use as an embedding for classification, and that conditions the first and second time step of computation :

$$u' \leftarrow MLP_u\Big(\Big[\sum_i X'_i || u\Big]\Big)$$

Prediction : the final step is passing the resulting vector u through a final multi-layer perceptron to produce logits for our binary classification problem :

$$out \leftarrow MLP_{out}(u')$$

We use the same dimensionality for the output vectors as for the input vectors of the message computation, node aggregation and graph aggregation, and this allows us to stack N GNN computations in a recurrent fashion.

c.1.3 Recurrent Deep Sets

We introduce a simpler model we term Recurrent Deep Sets (RDS). This model is introduced to provide a comparison point to the MPGNN and assess how useful relational inductive biases are in performing well on the benchmark. This method dispenses with the message computation and node aggregation part, and at each step only transforms the node features and aggregates them into the graph feature. This architecture is resembles the Deep Set, to the important difference that the graph-level feature u is then fed back at the following step by being concatenated to the object feature for the next round of computation. This allows the computation of features for each object to depend on the state of the whole configuration, as summarized in the graph embedding u. This contrasts with the original Deep Sets, where each object is processed independently. The functional description of this model is thus :

$$X'_j \leftarrow MLP_X([X_j || u])$$

$$u' \leftarrow MLP_u\Big(\Big[\sum_i X'_i || u\Big]\Big)$$

$$out \leftarrow MLP_{out}(u')$$

Note that for this model, there is no need to connect each object to every other object. However, this back-and-forth between node computation and graph aggregation can be interpreted as computing messages between each object and a central node, that represents the information of the whole graph. In this sense, this model can be interpreted as a GNN operating on the star-shaped graph of the union of the set of objects and the central graph-level node. In particular, this means that the resulting model performs a number of computations that scales linearly in the number of nodes, instead of quadratically as is the case for a message-passing GNN on the complete, fully connected graph of objects. While this is an interesting propriety, in practice for a fixed size of u the number of objects cannot grow arbitrarily large because the success of our models depend on the ability of u to accurately summarize information which is dependent on all the objects, which becomes difficult as the number n of objects becomes large.

c.1.4 Deep Sets

In this section we summarize shortly the computations done by the Deep Set model. The model can be described as a node-wise transformation composed with a sum operator on all the nodes, followed by a final transformation. Namely, the Deep Set defines the following transformations:

$$X'_j \leftarrow MLP_X(X_j)$$
$$u' \leftarrow \left[\sum_i X'_i\right]$$
$$out \leftarrow MLP_{out}(u')$$

Note that, contrary to the MPGNN and the RDS, the Deep Set has no recurrent structure; running it several times will always produce the same output.

C.1.5 Hyperparameters

In our experimental setup, for a MPGNN/RDS/Deep Set we let h be the dimension of the hidden layers for all internal MLPs (MLP_E, MLP_X, MLP_u, and MLP_{out}, when each of these MLPs are defined, as appropriate). We let d be the number of hidden layers. We then have, to keep a similar number of parameters between GNN models, $h = 16$ and $d = 1$ for MPGNN, $h = 16$ and $d = 2$ for RDS, and $h = 16$ and $d = 4$ in Deep Set. We use ReLU non-linearities in each MLP. We use (for MPGNN and RDS) $N = 1$ successive passes through the GNN, since increasing N did not seem to affect the performance in a significative way.

We also define the MLP baseline as having $d = 2$ layers of $h = n_{obj} \times 16$ hidden units. This was done to provide the MLP with a roughly comparable number of units to the GNNs (since the latter models maintain a hidden representation of size 16 for each node). The number of units here refer to the cumulative dimensions of the hidden vectors, the number of parameters to the number of scalar weights and biases. In particular, this design was adopted because the number of hidden units

C.2 Models for Discrimination

To tackle this task, we construct from one sample of two configurations two different graphs, one representing each set of objects, in the same way as in Identification. In this section we introduce a straightforward Dual-Input Model (hereby referred as DIM) that operates on input pairs of graphs. The internal GNNs used inside the DIM can be any one of MPGNN, RDS or Deep Set, and we will identify different dual-input models by their internal component type.

C.2.1 Dual-input architecture

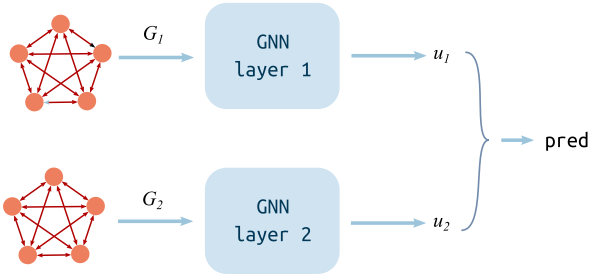

Figure S2. An illustration of the two dual-input architecture. Two parallel layers (MPGNN, RDS or Deep Set) process the input graphs in parallel, and the resulting global vectors are concatenated and passed through a final MLP.

Let us denote by GNN a GNN layer, as defined in the discussion of Discrimination architectures. The DIM is composed of two parallel GNN layers, GNN_1 and GNN_2. Each input graph is processed by its corresponding layer, as such:

$$X_1', E_1', u_1' \leftarrow GNN_1(X_1, A_1, E_2, u_1)$$
$$X_2', E_2', u_2' \leftarrow GNN_2(X_2, A_2, E_2, u_2)$$

As previously, we repeat this operation N times, and we produce the output as:

$$out \leftarrow MLP_{out}([u_1'||u_2'])$$

c.2.2 Hyperparameters

We use the same hyperparameters in for this task as in the previous one. The MLP baseline is also defined in the same way, except that the number of hidden units in each layer is doubled to account for the doubling in number of objects. Since the datasets used in Discrimination contain a variable number of objects across samples, we use the mean n_{obj} for determining the number of hidden units in the MLP.

D MODEL HEATMAPS

This section provides additional discussion on the model heatmap visualizations presented in the eponymous section in the main text. We present more fully the description of what these visualizations mean and we provide additional commentary on the qualitative differences between models, conditions (*low* number of objects, *mid* number of objects and *high* number of objects).

d.1 Additional details on heatmap generation

Each one of the models we use in this work projects the input graph $G = (X, A, E, u)$ on a two-dimensional vector with coordinates $(\mathcal{C}_+, \mathcal{C}_-) \in \mathbb{R}^2$. These values correspond respectively to the scores (logits) for the positive and the negative classes: if $\mathcal{C}_+ \geq \mathcal{C}_-$ the input is classified as positive, otherwise it is classified as negative. To produce one heatmap image for an object of index o_i of feature vector X_i, we plot $H = \mathcal{C}_+ - \mathcal{C}_-$ as a function of o_i's x-y position, while holding o_i's non-spatial features as well as all other object features constant. Thus, every pixel where H is positive corresponds to an input with an alternative x-y position for o_i that the model classifies as positive. The same thing holds for negative values of H: they correspond to positions of o_i that would result in the input being classified as a negative. The actual prediction of the model for the given input is given by the color of the current position of o_i, marked by a star in our plots.

In this section we plot the heatmaps for Discrimination models. We do this according to the previous description, by comparing a configuration with a copy of itself, and by moving an object in the copy configuration only; in this case o_i refers to one of the objects in the copied configuration.

d.2 Discussion

The heatmaps are given in Figure S3 and Figure S4 for different models, training datasets, numbers of objects and seeds. Looking at these model heatmaps allows us to have a qualitative grasp of the functions learned by our different models, and in particular how well these functions encode the similarity classes they are trained to represent. The Deep Set models were not included in the figures because these models predict the same value of H for each position of o_i. This means that, when holding the objects $o_j, j \neq i$, fixed, the model is (almost) invariant to changes in position of o_i.

Before going further, one should note that these plots allow us to visualize the variation of the models' learned function only with respect to two variables among many, and the portion of the variation we visualize becomes smaller as n_{obj} grows, because adding objects is adding variables. Nevertheless, these

variations are important because they allow us to probe the boundaries of what our models classify as being the same configuration as opposed to what they classify as being different configurations.

One of the first thing we can note is the qualitative difference between MPGNN and RDS models, espescially when n_{obj} is low (Figure S3). RDS heatmaps seem to consistently exhibit a ring-like structure, with the areas corresponding to the positive class form a ring centered around the center of the configuration and passing through o_i. We conclude from this that the model has leaned to use the distance from the center of the configuration (which has a good chance of being different for each object of a random configuration) as one of the main features in classifying its input. This is to be expected when we look at the computations done by the RDS: each node has access to an average of all the other nodes before performing its own node update. MPGNNs sometimes learn ringlike structures that seem more modulated as in the case of RDS, sometimes being open rings. Other times, MPGNNs heatmaps exhibit a kind of cross-like structure, or two symmetrical rings; o_i is placed at one of the high-value spots of this structure (indicating that the model has learned to assign the positive class to a set of two identical copies of the same configuration). These structure seem to exhibit symmetry with respect to the principal axis of the configuration, suggesting that MPGNN learns to compute and use this as a feature when tasked to compare two different configurations it never has seen before. The different forms of the trained MPGNNs may also hint at a higher expressivity of the model, its ability to approximate a wider range of functions.

Another interesting thing this visualization allows us to see is the difference in functions learned by models on two different datasets. Figure S3's second and third rows compare models on the same configuration of 8 objects, but the ones in the second row have been trained with $n_{obj} \in [3..8]$ whereas the ones in the third row have been trained with $n_{obj} \in [9..20]$. The function learned exhibit qualitative differences, even if the presented configuration and the models are he same, as a result of different training conditions. The heatmaps in the bottom row appear more spread out. We take it to show that the functions learned while training on higher numbers of objects are less sensitive to the variation of a single object's position. This is probably so because of the way the negative samples are created in our datasets: randomly resample all object positions (and then rotate, scale, and translate all objects randomly). As n_{obj} gets larger, the compared examples presented have a very high probability to be widly different from the target configuration, making the model less likely to learn about the contribution of the perturbation of only one object.

Figure S4 corroborates this view: the functions learned exhibit much less variation to the perturbation of the position of a single object, particularly in the *high* ($n_{obj} = 25$) case. This figure also showcases a prediction error: the top row of the bottom-right block is a visualization of an RDS model that assigns the negative class to all alternative positions of the object o_i, including its current one. This should not surprise us: when training with a *high* number of objects, many RDS models do not train and perform only slightly above chance.

E EASIER AND HARDER CONFIGURATIONS TO IDENTIFY

In this section we study why particular configurations may be harder or easier to recognize for our models, in the context of the Identification task. We hypothesise that more regular arrangements of objects must be easier to tell apart than more random configurations, and that configurations with a high degree of object diversity (many colors, many shapes) must also be easier to learn to classify, because the models can more easily identify and match the different objects. To test this, we compare one randomly generated dataset (regular difficulty) with 1) a configuration where all objects are red circles of the same size positioned at the same point; 2) a configuration where all the objects are red circles of the same size arranged in a line; 3) a configuration where all the objects are randomly positioned red circles of the same size; and 4)

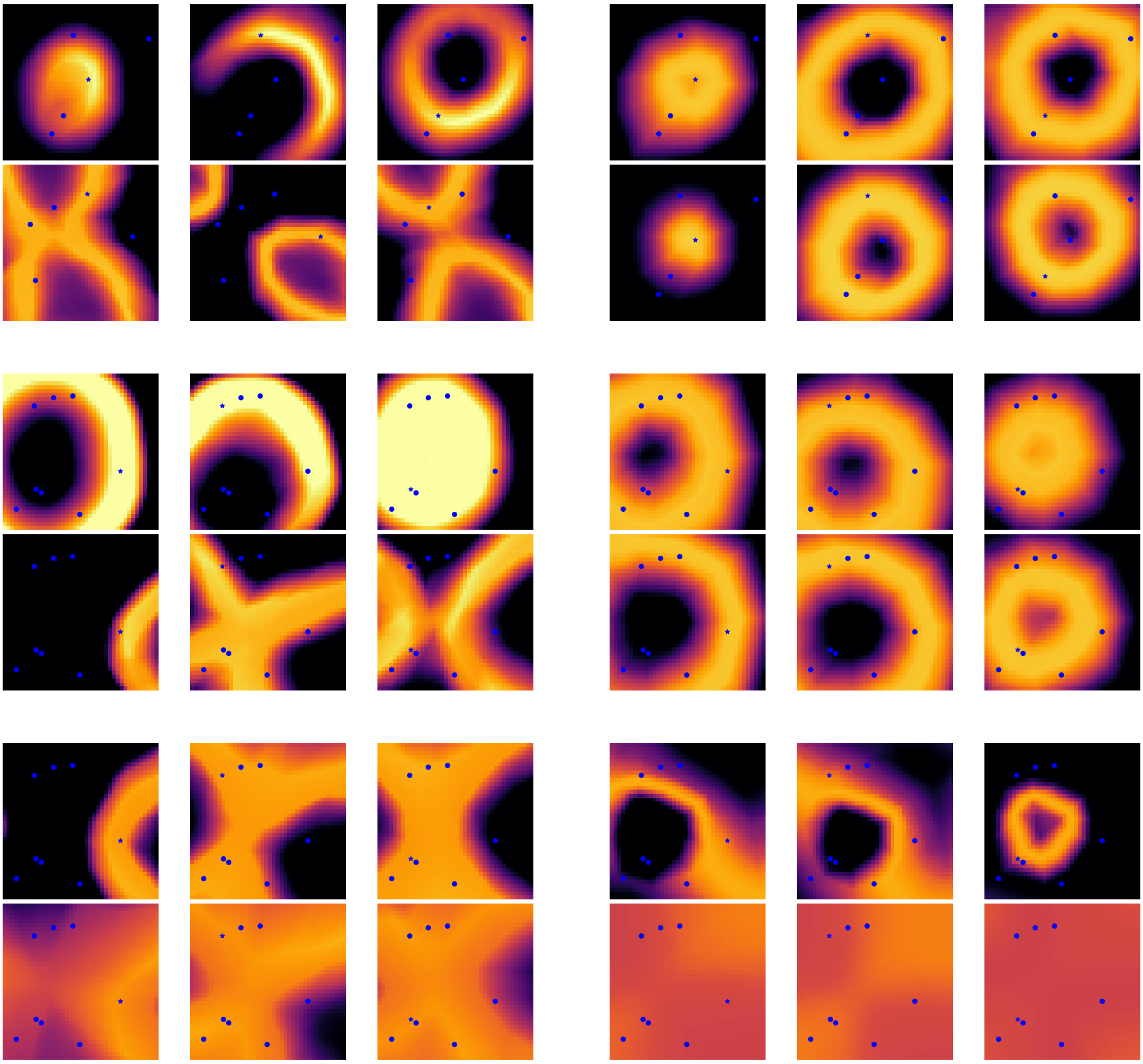

Figure S3. Model heatmaps for Discrimination models. The plots are organized as follows: the left column corresponds to dual-input models with MPGNN internal layers, the right one plots dual-input models with RDS layers. Each of the larger-scale rows plots, respectively: models trained on *low* numbers of objects ($n_{obj} \in [3..8]$) and 5 objects plotted, models trained on *low* numbers of objects and plotted with 8 objects, and models trained on *mid* numbers of objects ($n_{obj} \in [9..20]$) and plotted with 8 objects, for contrast. Within each of the six blocks, each three-image row corresponds to the heatmaps generated on one random training run of a model, and each image corresponds to moving about one particular object o_i. For each image, the fixed objects are represented by a blue dot corresponding to their position, and the perturbed object is identified with a blue star.

the same configuration as 3), but with circles of varying color. We train our three layers, DS, RDS and MPGNN, to recognize these configurations, and report the results in Figure S5, along with an illustration of the configurations.

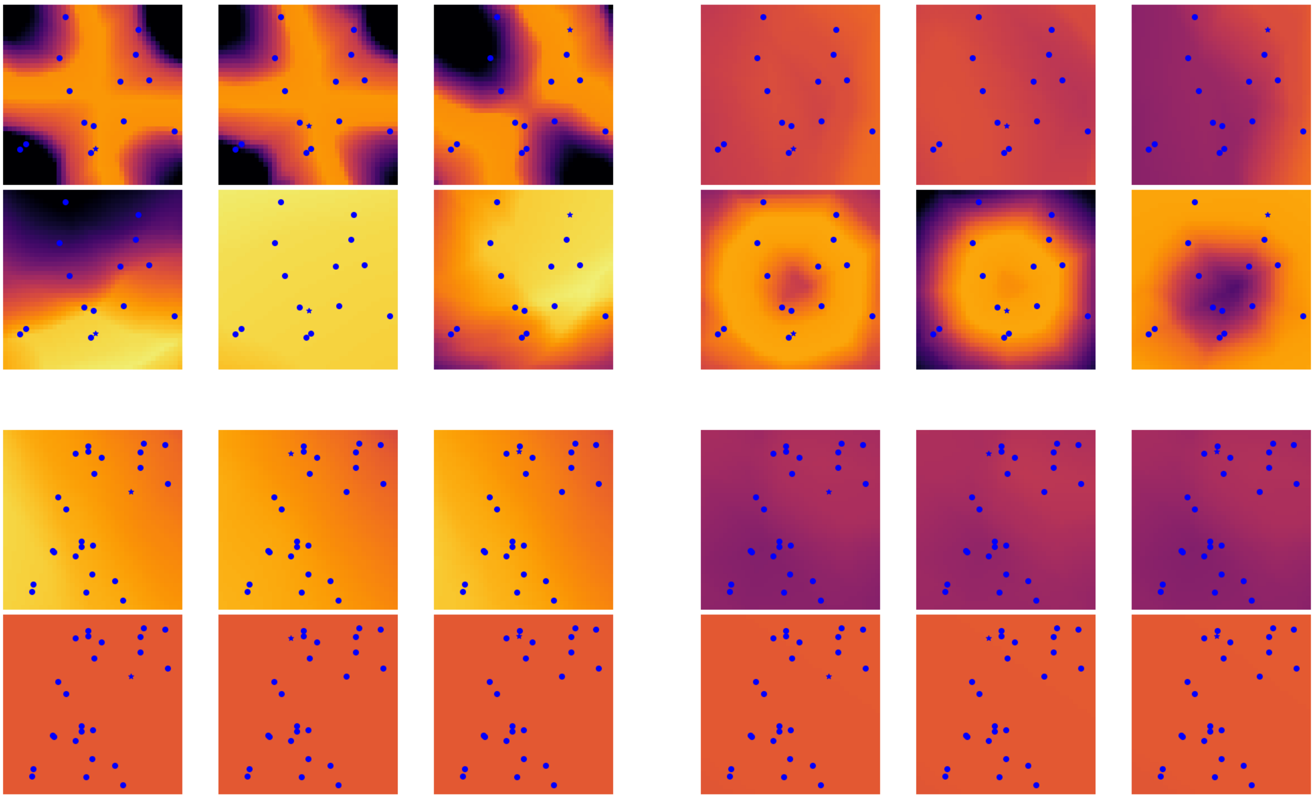

Figure S4. Model heatmaps for Discrimination models. The left and right columns are respectively MPGNN and RDS, as in Figure S3. The large-scale rows correspond to models trained on *mid* numbers of objects and plotted with a configuration of 15 objects, and models trained with *high* ($n_{obj} \in [21..30]$) numbers of objects and plotted with 25 objects.

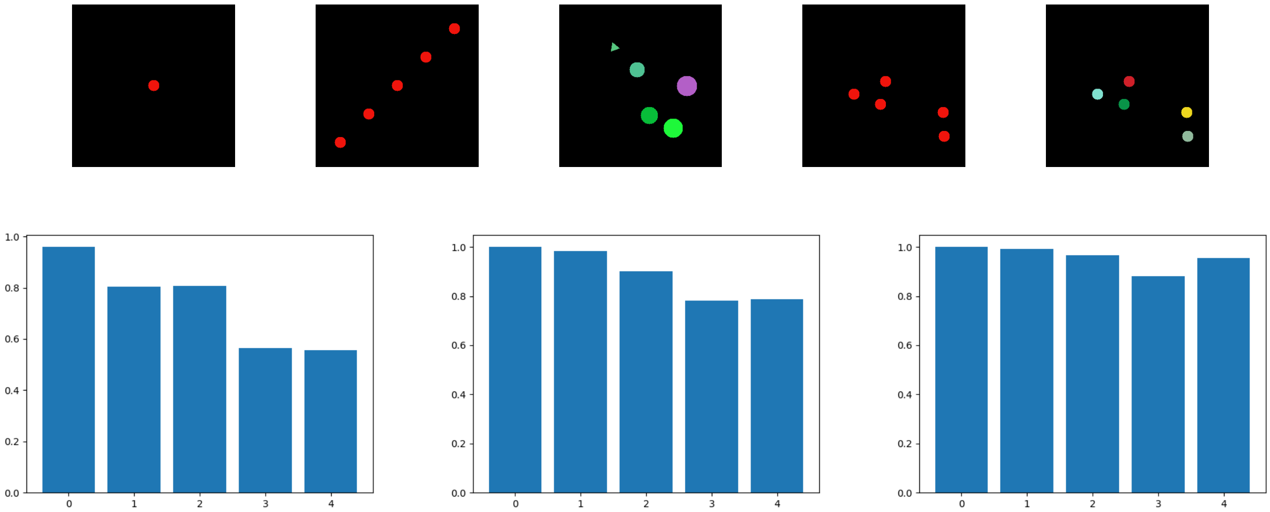

Figure S5. The top row represents the configurations we trained our models with, as described in the text. The bottom row is a bar plot of the final test accuracy of (from left to right) the Deep Set, Recurrent Deep Set and Message-Passing GNN on each of the 5 datasets, in the order specified in the top row (results were computed on 5 seeds for each dataset).

We interpret the results as follows : the fourth configuration, the one with all red circles, does seem to be more difficult to learn across all models. This may be due to the intrinsic hardness of the task on this configuration, or to the fact that randomly resampled positions for the negative examples of this dataset may give with non-negligible probability configurations that are close to a translated/rotated version of the reference example, because any object can be identified with any other. This second option may translate into negative examples that may resemble strongly positive examples, confusing the model. Interestingly, the problem fades when we identify each object by giving it a color, suggesting this second explanation is correct, but only for the MPGNN. For MPGNN, performance is roughly similar on all the other datasets. However, for DS and RDS, there seems to be considerable difference between datasets. The DS layer fails to perform significantly above chance for both right-hand configurations, suggesting arrangements of similar objects are difficult for this kind of model. Interestingly, the DS layer performs similarly on the aligned red circles than on the random diverse configuration, but significantly better than on the configuration with randomly scattered red circles, suggesting it is able to use the alignment information to reach above-chance accuracy, but not in a completely reliable way. As a contrast, the RDS layer performs near-perfectly on this configuration, showing that the additional connectivity of the RDS helps it in discovering exploitable regularities in the data.

F GENERALIZATION TO OTHER NUMBER OF OBJECTS

In this section we present some generalization results for the Discrimination task. Since the models for this task are trained on any couple of configurations, they can be transferred to datasets with higher numbers of objects. In this experiment we train Deep Set, RDS and MNGNN models on one dataset ($n_{obj} \in [3..8]$, $n_{obj} \in [9..20]$ or $n_{obj} \in [21..30]$) and test the models on all three datasets. The results are reported in Table f.

	3-8	9-20	21-30
3-8	0.51 ± 0.016<br>0.80 ± 0.133<br>**0.89** ± 0.03	0.49 ± 0.046<br>0.66 ± 0.138<br>**0.71** ± 0.092	0.50 ± 0.043<br>0.51 ± 0.048<br>**0.56** ± 0.075
9-20	0.51 ± 0.046<br>**0.75** ± 0.125<br>0.68 ± 0.063	0.50 ± 0.001<br>0.68 ± 0.154<br>**0.81** ± 0.121	0.50 ± 0.047<br>0.52 ± 0.054<br>**0.68** ± 0.16
21-30	0.50 ± 0.04<br>**0.60** ± 0.087<br>0.51 ± 0.048	0.51 ± 0.068<br>0.68 ± 0.15<br>**0.77** ± 0.12	0.50 ± 0.05<br>0.52 ± 0.04<br>**0.71** ± 0.18

Table S2. Generalization results between datasets for Deep Set, RDS and MPGNN. The numbers plotted are averages of testing accuracies. Columns correspond to training datasets, rows to testing datasets. Each block corresponds to one train-set/test-set combination. In each block, the results are given from top to bottom for Deep Set, RDS and MPGNN. Diagonal blocks correspond to matching train set/test set combinations. All reported results are averages and standard deviations over 10 different runs. Rows and columns are annotated with the n_{obj} range.

The results demonstrate the limited abilities of the models to transfer their learned functions to higher or lower numbers of objects. For instance, MPGNNs achieve 0.89 test accuracy when trained *and* tested on 3 to 8 objects, but this performance decreases sharply on the datasets with higher numbers of objects. This is less the case for RDS, presumably because the simpler functions they learn, while achieving lower performance when tested on the matching dataset, are more robust to higher numbers of objects. Another interesting point is that models trained on 9 to 20 numbers of objects appear to transfer better than other conditions. In particular, both RDS and MPGNN achieve higher mean test accuracy when transferring from

9-20 objects to 21-30 objects than models which were directly trained on 21-30 numbers of objects. The 21-30 dataset is harder to train on, so the models trained directly on this dataset may never learn, which bring the mean accuracy down. This suggests that functions useful for good performance on 9-20 numbers of objects are also useful for 21-30 numbers of objects. In contrast, functions useful for good performance on 3-8 numbers of objects do not transfer well to higher numbers of objects.

These suggest a tradeoff in being able to solve the task well for low numbers of objects versus being able to solve the task for high numbers of objects. This confirms the qualitative evaluation in Section d, where we remarked that the functions learned by the models varied greatly with the dataset they were trained on.

G TRAINING ON LESS EXAMPLES

In this section we vary the number of unique examples presented to the models in the training set. We keep the same number of optimizer steps as in the main experiments, but we reduce the number of samples we train on. The results for Identification are presented in Table 3, and the results for Discrimination are reported in Table 4.

In both Tables, in the first two rows we see all models overfitting the dataset, their test accuracy being at 0.5. They are unable to transfer to the training set and performing at chance levels. Then, respectively at 1000 samples for Identification and at 10000 samples for Discrimination the performance levels rise very close to their final levels. We wanted to observe whether the additional relational inductive biases in MPGNNs would allow for faster training than RDS and Deep Set; however we do not observe this: all models seem to have similar progression levels as the size of the training set increases. From this we conclude that the advantage of MPGNNs do not stem from their sample-efficiency, but rather from their ability to represent more complex functions.

	MPGNN	RDS	Deep Set
10	0.52 ± 0.038	0.52 ± 0.035	0.52 ± 0.032
100	$\mathbf{0.64} \pm 0.051$	0.58 ± 0.035	0.54 ± 0.019
1000	$\mathbf{0.94} \pm 0.041$	0.86 ± 0.065	0.61 ± 0.036
10000	$\mathbf{0.97} \pm 0.026$	0.91 ± 0.062	0.65 ± 0.079

Table S3. Mean accuracies for training on reduced numbers of examples on Identification. The last row represents the full training set.

	MPGNN	RDS	Deep Set
100	0.50 ± 0.005	0.50 ± 0.004	0.50 ± 0.005
1000	0.50 ± 0.004	0.50 ± 0.003	0.50 ± 0.005
10k	$\mathbf{0.87} \pm 0.016$	0.82 ± 0.098	0.52 ± 0.01
100k	$\mathbf{0.89} \pm 0.03$	0.80 ± 0.133	0.51 ± 0.014

Table S4. Mean accuracies for training on reduced numbers of examples on Discrimination. The last row represents the full training set.

H ADDING DISTRACTOR OBJECTS

In realistic environments cluttered with objects, only some of the objects could be relevant for the similarity task at hand; some of the objects may be distractors unrelated to the task. To test how robust our models

are to additional objects in the input that bear no relevance to the task, we generate additional train and test sets for $n_{obj} \in [3..8]$. We use numbers of distractors $n_d \in [0..3]$ for both Identification and Discrimination. The results are reported in Table S5.

Table S5. Test accuracies on the distractor datasets.

Model	Identificatiom	Discrimination
MPGNN	**0.87** $\pm$ 0.043	**0.76** $\pm$ 0.019
RDS	0.78 $\pm$ 0.102	0.59 $\pm$ 0.069
Deep Set	0.67 $\pm$ 0.073	0.51 $\pm$ 0.01

We see the model performance consistently drop for MPGNN and RDS, with a decrease in test accuracy of around 10% on both tasks. The distractors seem to have no effect on Deep Set performance, suggesting that Deep Sets do not rely on a precise representation of object configuration. Dealing effectively with distractor objects could be done by adding an attention mechanism to the GNNs, a topic we leave for further work.
